# Identification of a specific surface epitope of OmpC for *Escherichia coli* O157:H7 with protein topology facilitated affinity mass spectrometry

**DOI:** 10.1007/s00253-021-11511-8

**Published:** 2021-08-25

**Authors:** Wenbin Wang, Xinyue Zhou, Yunong Sang, Xiaxia Liang, Jianxin Liu, Saikun Pan, Luxin Wang

**Affiliations:** 1 Jiangsu Key Laboratory of Marine Bioresources and Environment, Jiangsu Ocean University, Lianyungang, Jiangsu China; 2 Co-Innovation Center of Jiangsu Marine Bio-Industry Technology, Jiangsu Ocean University, Lianyungang, Jiangsu China; 3 Jiangsu Key Laboratory of Marine Biotechnology, Jiangsu Ocean University, Lianyungang, Jiangsu China; 4 Department of Food Science and Technology, University of California Davis, Davis, CA 95618 USA

**Keywords:** *Escherichia coli* O157, Outer membrane protein C, Structure, Identification, Epitope

## Abstract

**Abstract:**

The goal of this work was to identify the target protein and epitope of a previously reported *Escherichia coli* O157:H7 (ECO157)–specific monoclonal antibody (mAb) 2G12. mAb 2G12 has shown high specificity for the recovery and detection of ECO157. To achieve this goal, the target protein was first separated by two-dimensional gel electrophoresis (2-DE) and located by Western blot (WB). The protein spots were identified to be the outer membrane protein (Omp) C by matrix-assisted laser desorption/ionization time-of-flight mass spectrometry (MALDI-TOF–MS). After that, the target protein was purified by immunoaffinity chromatography (IAC) and subjected to in situ enzymatic cleavage of the vulnerable peptides. Eight eluted peptides of OmpC identified by liquid chromatography–tandem mass spectrometry (LC–MS/MS) were further mapped onto the homologous protein structure of *E. coli* OmpC (2IXX). The topology of OmpC showed that three peptides had extracellular loops. Epitope mapping with overlapping peptide library and sequence homology analysis revealed that the epitope consisted of a specific peptide, “LGVING,” and an adjacent conservative peptide, “TQTYNATRVGSLG.” Both peptides loop around the overall structure of the epitope. To test the availability of the epitope when ECO157 was grown under different osmolarity, pH, and nutrition levels, the binding efficacy of mAb 2G12 with ECO157 grown in these conditions was evaluated. Results further demonstrated the good stability of this epitope under potential stressful environmental conditions. In summary, this study revealed that mAb 2G12 targeted one specific and one conservative extracellular loop (peptide) of the OmpC present on ECO157, and the epitope was stable and accessible on ECO157 cells grown in different environment.

**Key points:**

• *OmpC is the target of a recently identified ECO157-specific mAb 2G12.*

• *Eight peptides were identified from the OmpC by using LC–MS/MS.*

• *The specificity of mAb 2G12 is mainly determined by the “LGVING” peptide.*

**Supplementary Information:**

The online version contains supplementary material available at 10.1007/s00253-021-11511-8.

## Introduction

Immunoassays based on antibody-antigen reactions have been widely used to recover and detect foodborne pathogens, given their high sensitivity, automation, and simplicity (Valderrama et al. 2016). However, the specificity of such assays has been continuously challenged. Taking *Escherichia coli* O157:H7 (ECO157) as an example, cross-reactions of ECO157 monoclonal antibodies with bacterial species such as *Escherichia hermannii*, *Brucella melitensis*, and *Citrobacter freundii* have been reported (Law et al. 2015; Tokarskyy and Marshall 2008). One of the reasons for these cross-reactions is that the target antigen and the epitope of many previously identified antibodies remained largely unknown. Such missing information has limited the use of monoclonal antibodies and the development as well as improvement of the specificity of antibody-antigen-based immunoassays. Thus, there is an urgent need for additional studies to identify the target(s) of antibodies on the surface of bacteria.

Several strategies have been reported to study epitopes. Classic structural biology techniques like X-ray crystallography (Malito et al. 2014) have been reported to be the most accurate approaches as they can determine the interacting atoms between the antigen’s and antibody’s surfaces. However, there is no guarantee of success with these methods, because 1, only a small fraction of Ab–Ag complexes can be crystallized for epitope analysis (Lu et al. 2009), and 2, the X-ray crystallography relies on high degrees of sophistication and training (Opuni et al. 2018). Another common biological strategy is to construct an amino acid mutant library of the target protein(s) and translate and express them on yeast or phage (Kowalsky et al. 2015). While these mutagenesis methodologies can be accurate and powerful, local folding defects caused by mutation may affect the results. In addition, another limitation associated with these methods is the technical complexity of library construction and expression, which requires considerable expertise in molecular cloning (Najar et al. 2017). Peptide microarrays have also been used for studying epitopes. These methods rely on reactions between synthetic peptides and antibodies and are user-friendly. However, they are not cost-effective and are more suitable for linear epitope mapping rather than conformational epitopes (Forsström et al. 2014).

In the last three decades, mass spectrometry and enzyme digestion-based methods have been reported to be alternative approaches for rapid and robust epitope mapping (Casina et al. 2014; Lu et al. 2009). The immobilization of antibodies on a solid support for separation (e.g., Sepharose agarose, magnetic beads) and the resistance nature of antibodies to enzymatical proteolysis lay the foundation for the two most frequently used approaches: epitope excision and epitope extraction (Opuni et al. 2018). While epitope extraction is limited to the mapping of linear epitope, epitope excision is applicable to both conformational and linear epitopes (Parker and Tomer 2002). One representative epitope excision method is the affinity-based proteolytic excision, which can be done using lab-made IAC and general LC–MS/MS (Zhao et al. 2014) or MALDI-MS (Moise et al. 2011).

In early 2020, this research team reported a novel mAb 2G12 with high specificity for ECO157:H7. This antibody showed no cross reaction with 68 non-*Escherichia coli* O157 strains when the cell concentrations were at 6 Log, and no reaction with 82.35% of the non-O157 strains when the cell concentrations were at 8 Log. The tested non-ECOO157 strains, which did not have cross-reactions with mAb 2G12, included *E. coli* O121, O111, O103, O45, O26, O101, O86, O78, O60, O25, O9, O6, O2, *Shigella* Sp., *Citrobacter amalonaticus*, *Enterobacter cloacae*, *Campylobacter* Sp, *Aeromonas* Sp., *Vibrio* Sp., *Proteus* Sp., *Listeria monocytogenes*, *Staphylococcus aureus*, and *Bacillus subtilis*. Initial evaluations indicated that the target of this mAb could be an outer member protein (Omp) with a size of approximately 35 kDa (Wang et al. 2021).

Antibodies against the bacterial Omps recognize the extracellular part of the transmembrane protein (Hollingshead et al. 2018; Malito et al. 2014). Information about the structure and topology of Omps could reveal the location of extracellular peptides, the lipid bilayer, and the periplasm (Dobson et al. 2015). This information would be instructive for subsequent epitope mapping. Over the years, several structures and topologies have been identified from bacterial Omps, and many of them have a β-barrel architecture with short loops between strands on the periplasmic side and large extended loops on the extracellular side (Rollauer et al. 2015). With the increasing amount of protein structure available in the protein data bank (PDB) and the topology information available in the Orientations of Proteins in Membranes (OPM) database (Lomize et al. 2012), the structure and topology of Omps are now available for the identification of unknown epitopes (Gourlay et al. 2017).

**Fig. 1:**
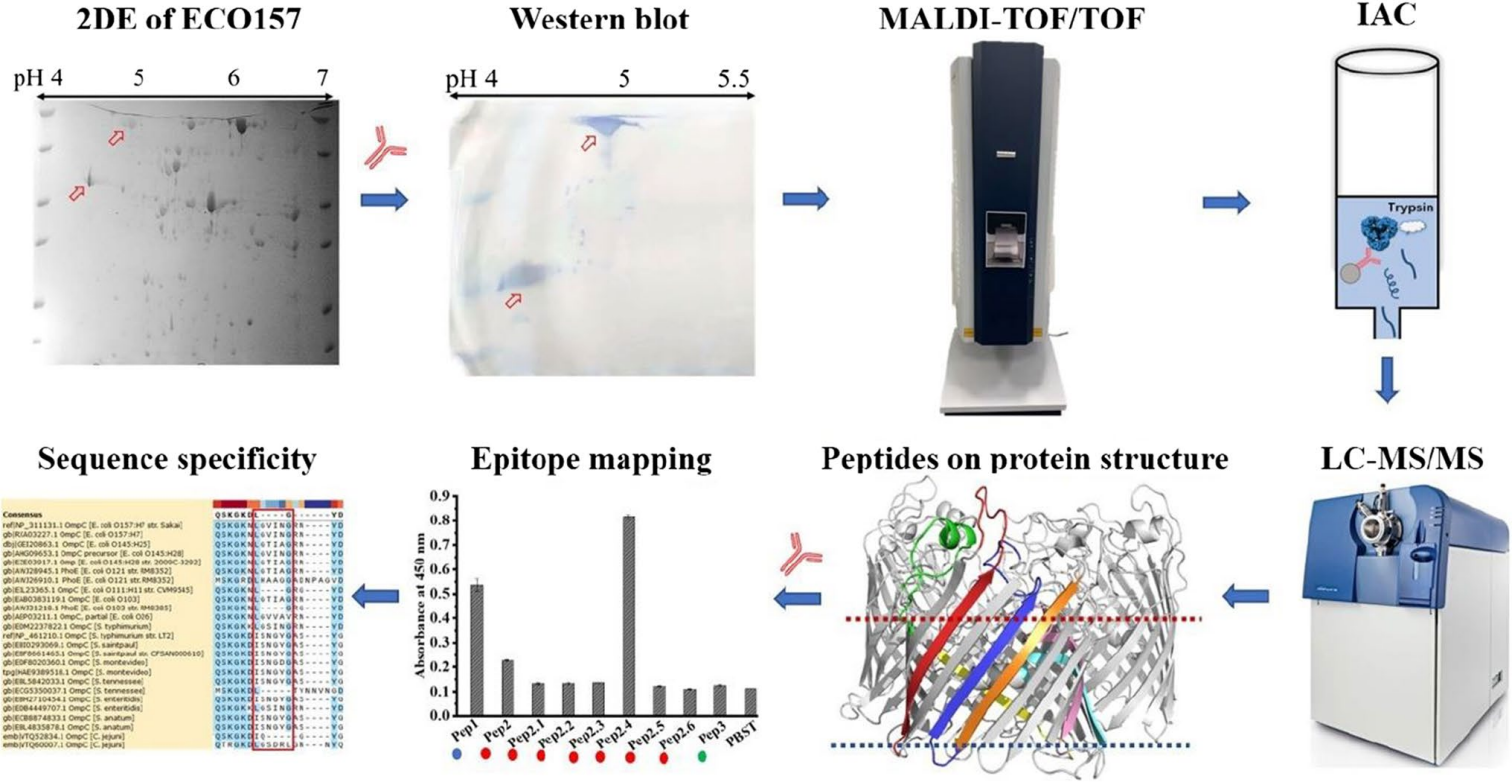
The workflow of the identification of ECO157-specific epitopes. Key steps include 2-dimensional electrophoresis (2-DE), matrix-assisted laser desorption/ionization time-of-flight/time-of-flight (MALDI-TOF/TOF), in situ enzymatic hydrolysis in immunoaffinity chromatography (IAC), liquid chromatography–tandem mass spectrometry (LC–MS), protein structure, membrane protein topology, sequence homology, and epitope mapping

The goal of this paper was to systematically investigate the target antigen and epitopes of this ECO157-specific mAb 2G12 and evaluate its availability and affinity with ECO157 under various simulated environmental stresses, e.g., osmolarity, pH, and nutrition levels. Figure 1 illustrates the key steps followed in this study. Two-DE, WB, and MALDI-TOF–MS were used for the separation, locating, and identification of the target Omp. IAC and in situ trypsin digestion were used for the purification of the target protein and the releasing of untargeted peptides. Eluted peptides of OmpC identified by LC–MS/MS were then mapped onto the protein structure of *E. coli* OmpC. Peptide libraries of the extracellular peptides and indirect enzyme-linked immunosorbent assay (ELISA) were used for epitope mapping. The sequence homology of the target OmpC extracellular peptides with large numbers of non-O157 strains and homologous OmpC structures were analyzed via the Basic Local Alignment Search Tool (BLAST) for the confirmation of the mAb 2G12 specificity.

## Materials and methods

### Main reagents and instruments

Iodoacetamide (IAA), dithiothreitol (DTT), goat anti-mouse IgG, trypsin, trifluoroacetic acid, 3-[(3-Cholamidopropyl) dimethylammonio]-1-propanesulfonate hydrate (CHAPS), NeutrAvidin protein, acetonitrile (ACN), trifluoroacetic acid (TFA), methanol, N,N-dimethylformamide (DMF), gelatin, ProClin 300, 3,3′,5,5′-tetramethylbenzidine (TMB), and sulfuric acid were purchased from Sigma-Aldrich (St. Louis, MO, USA). Pharmalyte 4–6.5 (high-resolution amphoteric electrolyte) for isoelectric focusing (IEF), immobilized pH gradient (IPG) strips (pH 4–7), IPG buffer pH 4–7, mineral oil, 2-D clean-up kit protein purification kit, 2-D quant-kit protein quantitative kit, polyvinylidene difluoride (PVDF) membrane, Ettan™ IPGphor 3 solid-phase pH ladder, degree isoelectric focusing instrument, Ettan™ DALTsix vertical electrophoresis instrument, and TE 70 semi-dry transfer system were from GE Healthcare (Chicago, IL, USA). ABI 5800 MALDI-TOF/TOF Plus mass spectrometer was from Applied Biosystems (Foster City, CA, USA). Gel imager was from Bio-Rad ChemiDoc (Hercules, CA, USA). Multi-Mode Reader Cytation5 was from Biotek (Winooski, VT, USA). Skim milk was purchased from BD (Lake Franklin, NJ, USA). Goat anti-mouse IgG was obtained from Jackson ImmunoResearch Inc. (West Grove, PA, USA). TMB substrate for Western blot was provided by GBCBIO Technologies Inc. (Guangzhou, China). High binding 96-well plates were purchased from Greiner Bio-one (Frickenhausen, Germany). NaHCO_3_, NaCl, Tris-Base, NH_4_HCO_3_, Na_2_CO_3_, Tween 20, and acetic acid were obtained from Sinopharm Chemical Reagent Co., Ltd. (Shanghai, China).

### Preparation of protein samples

Three *Escherichia coli* O157:H7 strains (CICC 21,530, ATCC 35,150, and ATCC 700,728) were obtained from the China Center of Industrial Culture Collection (CICC) and American Type Culture Collection (ATCC). Cultures were activated in Luria–Bertani (LB) broth for overnight at 37 ℃. After centrifugation at 5,000 g and 4 ℃ for 10 min, cells recovered from 100 mL of liquid culture were resuspended in 10 mL of fresh bacteria cell lysis buffer (8 M urea, 4% chaps, 2% IPG buffer, 40 mM DTT). Whole protein extract was prepared by an ultrasonic crusher (LC-CS950, Tocan, Shanghai, China). Briefly, the sample was ultrasonicated at 4 ℃ in an ice bath at 475 W for 20 min with a 9.9-s interval after every 5 s of sonication. The supernatant was collected after centrifugation at 12,000 g and 4 ℃ for 10 min. To purify the protein, trichloroacetic acid (TCA)-acetone (10%) pre-cooled at − 20 ℃ (40 mL) was added with a volume ratio of 5:1 to precipitate the protein overnight at − 20 ℃. The precipitation was collected by centrifugation at 12,000 g and 4 ℃ for 10 min. After that, 20 mL of pre-cooled acetone was added and mixed. The precipitation was collected again by centrifugation at 12,000 g and 4 ℃ for 15 min, and this protein precipitation process was repeated twice. After air-drying at 25 ℃, the precipitation was added with 5 mL of sample lysate and incubated in a water bath at 30 ℃ for 1 h to fully dissolve the protein. After centrifugation at 12,000 g for 10 min, the supernatant was collected and centrifuged again to fully remove incompatible impurities. The supernatant sample of ECO157 CICC 21,530 was further purified and quantified by a 2D clean-up kit and a 2D quant-kit according to the instructions of the manufacturer (GE Healthcare). The supernatant sample of ECO157 ATCC 700,728 was prepared the same way and dialyzed with 10 mM of PBS for 24 h. After centrifugation at 12,000 g for 10 min, the supernatant was collected and quantified by the Bradford assay (Compton and Jones 1985) before being used for IAC. The cell lysate of ECO157 ATCC 35,150 was analyzed by gel electrophoresis (SDS-PAGE) and Western blot. The SDS-PAGE gel was aligned with the Western blot and the target protein band (stained by Coomassie blue) was cut, decolorized, and subjected to in-gel digestion and the following analysis by the LC–MS/MS.

### Two-dimensional gel electrophoresis

Two-dimensional gel electrophoresis (2-DE) was conducted following the protocol in the General Electric (GE) manual. One hundred seventy microliters (2.5 mg) of ECO157 protein was mixed with an equal volume of hydration solution (8 M urea, 2% CHAPS, 0.5% IPG buffer, 0.002% bromophenol blue), and then added into the isoelectric focusing hydration tank (GE Healthcare, 18 cm). The IPG strip (PH 4–7 L, 18 cm) was then immersed in the sample buffer and covered with mineral oil (2.3 mL). IEF was performed according to the following steps: (1) Stp (one step voltage rise) 30 V 12 h, (2) Stp 200 V 1 h, (3) Stp 500 V 1 h, (4) Grd 1000 V 1 h, (5) Grd (gradient voltage rise) 4000 V 1 h, (6) Grd 8000 V 1 h, (7) Stp 8000 V 100,000 Vhr. The IPG strip was then taken to a tank and incubated with the equilibrium solution (6 M urea, 30% glycerol, 2% SDS, 0.002% bromophenol blue, 75 mM Tris–HCl, pH 8.8) containing 2‰ (m/v) DTT (10 mL) for 15 min. After that, it was added with the equilibrium solution containing 3% (m/v) IAA (10 mL) and incubated for an additional 15 min. The 10% separating SDS-PAGE gel (18 cm16 cm1.5 mm) was prepared following instructions of the manufacturer (GE Healthcare). Vertical electrophoresis was conducted in two steps: step 1: 600 V, 400 mA, 2 W/gel, and step 2: 600 V, 400 mA, 20 W/gel. After electrophoresis, the gel was added with 60 mL of 12.5% trichloroacetic acid and incubated for 30 min. The gel was stained with 60 mL of 0.1% Coomassie brilliant blue G-250 for 10 min. Then, the gel was added with 60 mL of 1% acetic acid with 1% methanol. The buffer was replaced every 3–5 h until the background of the gel was completely detained.

### Western blot

WB was conducted following the protocols described by Wang et al. (2020). To better fit the TE 70 semi-dry transfer system (14 cm16 cm), the 2-DE gel (18 cm16 cm) was cut into four parts (9 cm9 cm1.5 mm2, two 9 cm7 cm1.5 mm2) and transferred onto PVDF membrane (9 cm9 cm1.5 mm2, two 9 cm7 cm1.5 mm2) individually. Each gel was placed on the PVDF membrane (immersed in 10 mL of methanol for 15 s before use) that was supported with two layers of filter paper pre-immersed in the anode buffer I (300 mM Tris–HCl, 10% methanol, pH 10.4) and one layer of filter paper pre-immersed in anode buffer II (25 mM Tris–HCl, 10% methanol, pH 10.4). Another three layers of filter paper pre-immersed in cathode buffer (25 mM Tris–HCl, 40 mM glycine, 10% methanol, pH 9.4) were then attached to the top of the gel. The layers were then put in the semi-dry transfer system and the protein was transferred at 25 mA for 30 min. The PVDF membrane was then immersed in 10 mL of methanol for 10 s and blocked with 30 mL of 5% skim milk in 10 mM of PBS (pH 7.2) at 4 ℃ overnight. Between each WB step, membranes were washed four times in 15 mL of 10 mM phosphate-buffered saline with tween 20 (PBST) for 3 min of gentle shaking. Fifteen microliters of the antibody 2G12 (0.3 μg/mL) in PBST was added and incubated with the PVDF membrane at 25 ℃ for 1 h. The preparation and detailed message of mAb 2G12 was described in our previous work (Wang et al. 2021). Fifteen microliters of the goat anti-mouse IgG antibody (0.2 μg/mL) in PBST was added and allowed to react at 25 ℃ for 1 h. After four times of washing as described above, 10 mL of the commercial TMB substrate for Western blot (GBCBIO, Guangzhou, China) was added and allowed to react for 5 min at 25 ℃. The reaction was stopped by replacing the substrate in 15 mL of double-distilled water for 20 s, and pictures were captured with a gel imager (Bio-Rad ChemiDoc, Hercules, CA, USA).

### In-gel digestion and MALDI-TOF/TOF

After WB, target gel spots on the Coomassie blue–stained 2-DE and SDS-PAGE were taken and digested by trypsin as described by Katayama et al. (2001). Briefly, gel spots were transferred into 0.5-ml centrifuge tubes and rinsed twice with 300 μL of ultrapure water. Three hundred microliters of 25 mM of NH_4_HCO_3_ in 50% (V/V) ACN was added to decolorize the gel dots for 30 min. The gel dots were then rinsed with 300 μL of double-distilled water two times. The double-distilled water was aspirated and added with 300 μL of 50% (V/V) ACN to dehydrate for 30 min. The buffer was replaced with 300 μL of 100% (V/V) ACN and the gel dots were dehydrated for another 30 min. The buffer was replaced with 10 μL of 25 mM NH_4_HCO_3_ and dots were allowed to swell for 30 min. Trypsin (0.4 μg) in 200 μL of 25 mM NH_4_HCO_3_ was then added and incubated in a water bath at 37 ℃ for overnight. The supernatant was transferred into new 0.5-ml centrifuge tubes, and the remaining gel was added with 50 μL of 5% TFA in 67% ACN and incubated at 37 ℃ for 30 min to extract the remaining peptides. After centrifugation at 5,000 g for 5 min, the peptide extracts and the supernatant of gel spots were combined and air-dried at 37 ℃. A gel particle with the comparable size of the protein spots was cut from the unstained part of the gel and used as the negative control.

For MALDI-TOF/TOF analysis, two samples and the control were resuspended with 5 μL of 0.1%TFA followed by mixing in 1:1 ratio with a matrix consisting of a saturated solution of α-cyano-4-hydroxy-trans-cinnamic acid in 50% ACN with 0.1% TFA. One microliter of the mixture was then spotted on a stainless-steel sample plate. Three replicates were done for each sample. Peptide MS and MS/MS were performed on an ABI 5800 MALDI-TOF/TOF Plus mass spectrometer and the data was acquired in a positive MS reflector using a CalMix5 standard to calibrate the instrument (ABI5800 Calibration Mixture). Both the MS and the MS/MS data were integrated and processed by using the GPS Explorer V3.6 software (Applied Biosystems, USA) with default settings. Based on the combined MS and MS/MS spectra, proteins were successfully identified based on a 95% or higher confidence interval of their scores in the MASCOT V2.3 search engine (Matrix Science Ltd., London, UK). The following search parameters were used: *E. coli* O157 as target strain; trypsin as the digestion enzyme; one missed cleavage site; fixed modifications of carbamidomethyl (C); partial modifications of acetyl (protein N-term), deamidated (NQ), oxidation (M); 100 ppm for precursor ion tolerance and 0.4 Da for fragment ion tolerance. Two trials of the experiments were conducted and three replicates of each sample were tested for protein identification in each trial.

### Affinity chromatography and LC–MS/MS

The affinity chromatography column was prepared as described by Graeber and Korkhov (2020). Three hundred milligrams of cyanogen bromide (CNBr) Sepharose agarose was swelled with 3 mL of 1 mM HCl solution for 15 min and washed with coupling buffer (0.1 M NaHCO_3_, 0.5 M NaCl, pH 9.0). After centrifugation at 1,500 g for 2 min, the supernatant was discarded, and 2 mL of *E. coli* O157: H7 mAb 2G12 (1 mg/mL) in coupling buffer was added to resuspend the Sepharose. Samples were then rotated at 25 ℃ for 2–4 h. An OD_280_ value of the supernatant at less than 1/10 of the original OD_280_ value indicated the endpoint of coupling. After three repeated washing processes with 3 mL of coupling buffer and centrifugation at 1,500 g for 2 min, the supernatant was discarded and 2 mL of blocking solution (1 M Tris-Base in coupling buffer) was added and incubated with shaking at 25 ℃ for 2 h. After centrifugation, the agarose was retained and resuspended in 2 mL of coupling buffer. After that, 0.9 mL of agarose solution was added to the vertically placed column, and was let to stand still for 10 min to fully sink the agarose to the bottom. An upper sieve plate was installed, and the column was washed with 10 mL of 10 mM PBS. Finally, the columns were added with 1 mL of 10 mM PBS with 0.01% ProClin 300 (preservative) and stored at 4 ℃ before use. Based on the amount of Ab used for two columns (2.0 mg), the conjunction rate of Ab to the Sepharose (90%), and the volume of agarose solution for one column (0.9 mL), it is estimated that there was approximately 0.8 mg of mAb 2G12 in each column.

In situ enzymatic hydrolysis in IAC was used to capture the target protein and purify the epitope (Iuraşcu et al. 2016). The two columns prepared above were washed with 20 mL of 10 mM PBS before loading the samples. Fifty milliliters of the prepared *E. coli* O157: H7 ATCC 700,728 protein sample (0.5 mg/mL in PBS) was slowly loaded to the columns (10 mL per column) at 30 ℃ and passed through the columns by gravity. After washing with PBST, 260 μg (Group A) and 520 μg (Group B) of trypsin in 2 mL of 25 mM NH_4_HCO_3_ were added respectively to the substrate of the two columns (bottom sealed) and incubated at 37 ℃ for 30 min. The columns were then washed with PBST again to rinse the free peptides. Bounded peptides were eluted with 2 mL of 0.1% TFA, 2 M acetic acid, and pure methanol. Elutes from the same column were collected and combined as one sample.

Eluted samples were then centrifuged at 12,000 × *g* for 10 min, and the supernatant was dried by the centrifugal concentrator and kept frozen at − 20 °C before the test. MS analysis was performed using the SCIEX’s TripleTOF 5600 LC/MS system. Peptide samples were dissolved in 5 μL of 0.1% formic acid and added into the C18 column (5 µm, 5 × 0.3 mm) by autosampler and then eluted to the analytical column (75 μm × 150 mm, 3 μm particle size, 100 Å pore size, Eksigent). Two mobile phases (mobile phase A: H_2_O, 0.1% formic acid and mobile phase B: ACN, 0.1% formic acid) were used to establish a 30-min gradient (0 min in 5% B, 15 min of 5–35% B, 1 min of 35–80% B, 80% B for 5 min, 0.1 min of 80–5% B, 5% B for 8.9 min). The flow rate of the liquid phase was 300 nL/min. In the mass spectrometer information-dependent acquisition (IDA) mode, each scan cycle contained an MS full scan (m/z range is 350–1500, ion accumulation time 250 ms), followed by 40 MS/MS scans (m/z range is 100 − 1500, ion accumulation time 50 ms). The MS/MS data was acquired when the precursor ion signal was greater than 120 cps and the charge number was + 2 ~  + 5. The exclusion time for repeated ion collection was 18 s. The mass spectrometry data generated by TripleTOF 5600 was retrieved by ProteinPilot (V4.5), and the database search algorithm was Paragon. The database used for searching was the proteome reference database of *E. coli* O157:H7 in UniProt. Two trials of the experiments were conducted with three replicates of each sample tested for LC–MS/MS in this trial.

### Protein structure, topology analysis

The protein structures and sequences of *E. coli* OmpC (2IXX, 2J1N) and *Salmonella* Typhimurium (3UPG) were obtained from the PDB. The topology information of OmpC (2IXX) was also available in the OPM database. The OmpC structure was analyzed by PyMOL, and the eluted peptides of OmpC after IAC and LC–MS/MS were labeled with different colors on the structure of *E. coli* OmpC (2IXX), which has high homology (query cover 94%, percent identity 92.37%) with the OmpC of ECO157. After comparison of the topology of OmpC on the cell membrane and locations of the eluted peptides on OmpC, peptides located on the extracellular loops were chosen for further analysis of the epitope.

### Epitope mapping and sequence homology analysis

The peptides of loop1 (TQTYNATRVGSLG), loop2 (QSKGKNLGVINGRNYD), and loop3 (YKINLLDDNQFTRD) and overlapping peptide library of loop2, which consists of six peptides with peptide length at 6 and offset at 2, were synthesized and purified by HPLC (> 90%) to further study the exact epitopes of mAb 2G12. The detailed peptide library of loop2 was synthesized because it has a much higher precursor signal (1269.363) in LC–MS/MS and is more accessible on the OmpC structure than other OmpC peptides. Loops 1, 2, and 3 were named according to the clockwise manner of the three adjacent long peptides with extracellular loops. All peptides were modified with a spacer arm of -SGSG- and a recognition molecule of biotin at the N terminal. The protocol of epitope mapping was conducted by indirect ELISA as described by Heuzenroeder et al. (Heuzenroeder et al. 2009) with minor revision. Briefly, the peptides were completely dissolved with a proper solvent like H_2_O (Pep2, Pep2.3, Pep2.6), 10 mM PBS (Pep3, Pep2.1, Pep2.2, Pep2.4) and DMF (Pep1, Pep2.5) and stored at − 80℃. Each well of the 96-well plate was filled with 50 μL of NeutrAvidin protein (3 μg/mL in 10 mM PBS) and kept at 37 °C for 2 h. The plates were washed 3 times with 10 mM of PBST (100 μL/well). This washing process was also conducted between each step of the ELISA protocol. One hundred microliters of 0.2% gelatin in 10 mM carbonate-bicarbonate buffer was then added to each well to block the non-specific reactions overnight at 4 ℃. After blocking, peptides were diluted to 500 μg/mL with PBST and added to each well (50 μL/well), and incubated at 4 ℃ overnight. Fifty microliters of mAb 2G12 (3 μg/mL) in PBST was added to each well of the plate and incubated at 25 ℃ for 1 h on an orbit shaker. Each well was added with goat anti-mouse IgG (0.7 μg/mL, 50 µL/well), covered by a lid, and incubated at 25 ℃ for another hour. TMB substrate (50 μL/well) was added to the plate and incubated at 37 °C for 15 min. Twenty-five microliters of 2 M of sulfuric acid was added into each well before reading the absorbance at 450 nm with the Multi-Mode Reader Cytation5 (Biotek, Winooski, VT, USA).

The sequence homology of the three extracellular loops was studied by BLASTp in NCBI. The query sequence (QEQ39178.1) was the OmpC of ECO157 and the subjected organisms were the tested strains including different *E. coli* serotypes that showed no cross-reaction with mAb 2G12. The homology of the three extracellular loops with all non-O157 strains was also studied in the same way. The parameter of the database was all the non-redundant protein sequences. For all non-O157 strains, the OmpC sequences matched a strain name at the species level were analyzed. The results of multi alignment were saved as aln files and visualized by SnapGene Viewer (V4.2.11).

### The availability of epitopes for mAb 2G12 under different growth conditions

ECO157 grew under different osmotic pressure, low nutrition, and pH levels were prepared for testing the availability of the epitope with mAb 2G12 (4 mg/mL) (Desai and Kenney 2019; Hahm and Bhunia 2006). ECO157 (LJH643, a clinical isolate obtained from a cantaloupe outbreak was kindly provided by Dr. Linda J. Harris at UC Davis) was first activated on LB agar. The single colony was inoculated to buffered peptone water (BPW) and cultured at 37 °C for 12 h. This freshly prepared culture was then added into fresh buffered peptone water (BPW) with different salt concentrations (1, 2, or 4%) or pH values (pH of 7.2 or 4.3 adjusted by HCl) with a ratio of 1% (v/v). To mimic a condition with limit nutrient, 0.1% (m/v) peptone water was used. The inoculated broth was incubated at 37 °C for 24 h. One hundred fifty microliters of the inoculated medium in each group was added in a microplate with three replicates and the growth curves were automatically collected by measuring the OD 600 nm in Cytation5 for 23 h. The concentrations of the culture were also obtained by plating onto plate count agar. The cells were collected by centrifugation at 5,000 g and 4 °C for 10 min, and resuspended in 10 mM of PBS. The binding between mAb 2G12 and ECO157 was measured by indirect ELISA with the same coating concentration of ECO157 at 7.0 log_10_ CFU/mL. All the results were obtained with three replicates in each group.

## Results

### Identification of the target protein of mAb 2G12

**Fig. 2:**
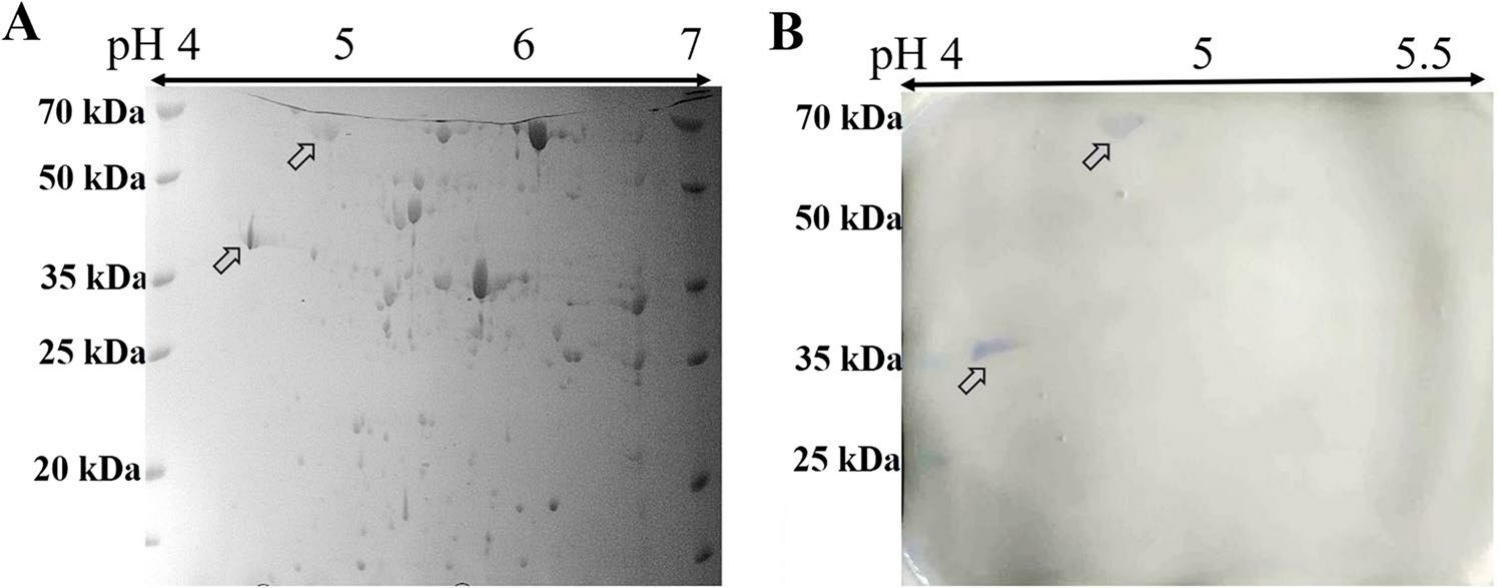
**A** Two-dimensional electrophoresis (2-DE) of *E. coli* O157: H7 whole-cell protein strained by Coomassie brilliant blue. **B** Western blot of mAb 2G12 with the 2-DE gel

Figure 2A shows the 2D image of the protein purified from the cell lysates of ECO157 (CICC 21,530). The protein dots were well separated according to molecular weight (MW) and isoelectric point (PI). The Western blot results of the four divided parts of two-dimensional gel electrophoresis (2-DE) showed that mAb 2G12 reacted with two protein spots, with the sizes being approximately 35 kDa (pI at around 4.5) and 70 kDa (with pI at around 5.0) on the left-upper part (Fig. 2B). The other three parts resulted in no protein spot and were not imaged. The results of in-gel digestion and MALDI-TOF/TOF mass spectrometry showed that the approximately 35 kDa protein dot was identified to be OmpC (40.48 kDa, pI 4.5), and the approximately 70 kDa protein dot was pinpointed as chaperone DnaK (69 kDa, pI 4.8) (Supplemental Figure S1).

### Peptides revealed by IAC, LC–MS/MS, and protein structure analysis

IAC column prepared with mAb 2G12 was used to purify OmpC from the whole proteins of ECO157 lysates and the target peptides after in situ enzymatic hydrolyses. Two groups with different amounts of trypsin were optimized for protein hydrolysis in the column. The trypsin (24 kDa) of 260 μg (group A) and 520 μg (group B) was chosen to mimic the theoretical molecular ratio of trypsin to OmpC (40.48 kDa) at 1:1 and 2:1. The amount of OmpC was calculated based on the conjugated amount of mAb in each column (0.8 mg/column) at the optimal condition when one antibody bound with two OmpC (0.43 mg/column). As shown in Table 1, group A with 260 μg of trypsin added resulted in 24 peptides (24 spectra) detected from a major outer membrane lipoprotein (8.3 kDa) with a protein score of 37.48, and 13 peptides (14 spectra) detected from OmpC with a protein score of 27.46. The protein score ranked second among all identified proteins. Group B with 520 μg of trypsin added resulted in eight peptides (nine spectra) detected from OmpC with a protein score of 17.58, which ranked first among identified proteins. The major outer membrane lipoprotein (protein score 16.14) ranked second and has eight peptides (eight spectra). The detailed sequences and information of the eluted OmpC peptides in groups A and B are provided in Supplemental Tables S1 and S2. No DnaK peptides were detected because cell proteins of O157 strain ATCC 700,728 were used in the affinity spectrometry to avoid the interference from DnaK.

**Table 1:** The identified proteins of ECO157 by IAC and LC–MS/MS with 260 μg trypsin (group A) or 520 μg trypsin (group B) added

Group	*N*	Score	Accession	Gene	MW (kDa)	Peptides (95%)	Spectra
A	1	37.48	P69778	*lpp*	8.323	24	24
	2	27.46	Q8XE41	*ompC*	40.508	13	14
	3	26.56	P0A911	*ompA*	37.201	18	20
B	1	17.58	Q8XE41	*ompC*	40.508	8	9
	2	16.14	P69778	*lpp*	8.323	8	8
	3	8.74	P0AED2	*uspA*	16.066	6	6

**Fig. 3:**
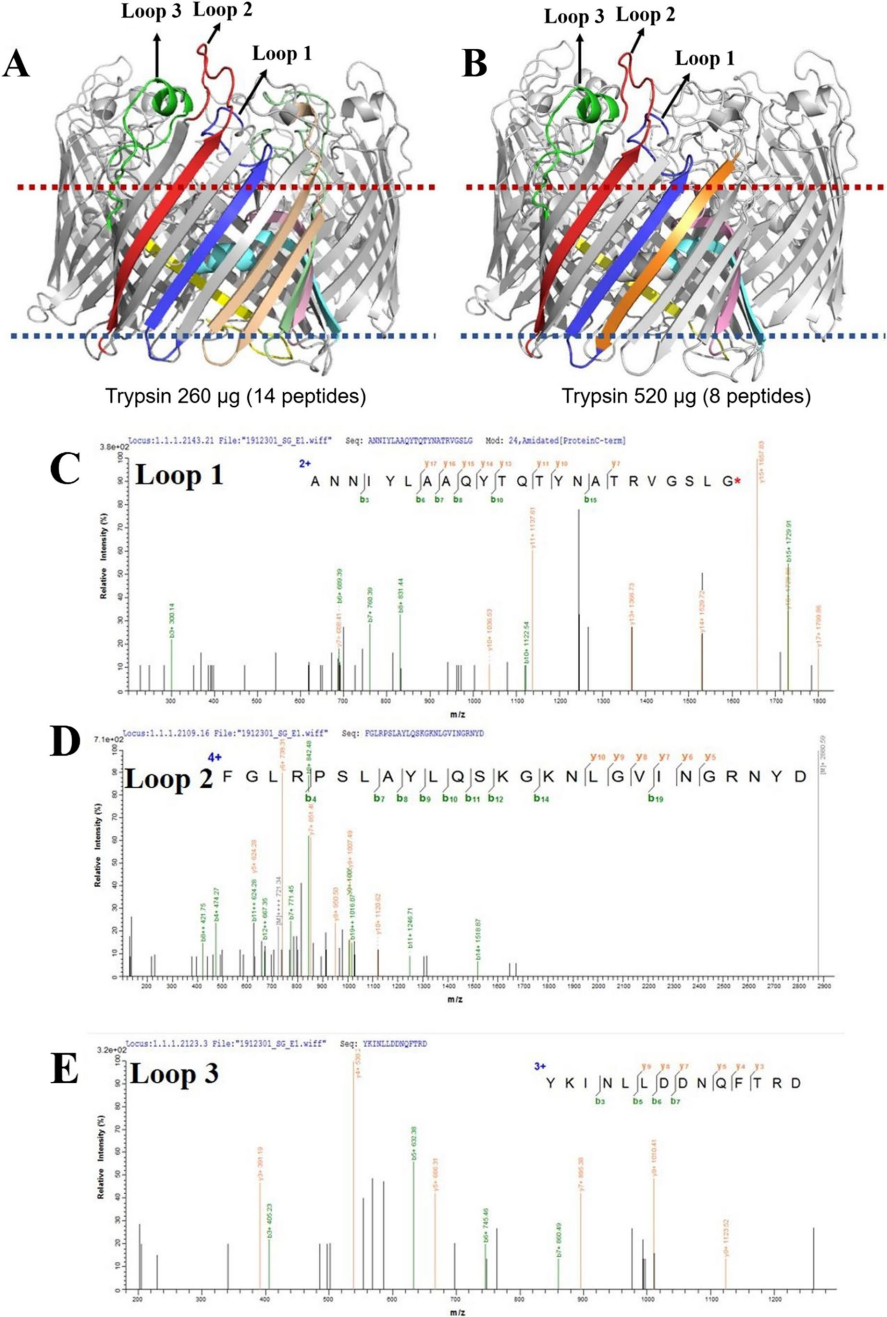
**A** The location of 14 peptides identified on *E. coli* OmpC (2IXX) in group A; **B** the location of eight peptides identified on *E. coli* OmpC (2IXX) in group B; **C**–**D** the mass spectrogram of peptide with extracellular loop1 (**C**), loop2 (**D**), and loop3 (**E**). Loops 1, 2, and 3 are labeled with blue, red, and green color respectively in Fig. 2A and B. In Fig. 2C–E, “FGLRPSLAYLQSKGKNLGVINGRNYD” is 286-311aa and is indicated with red signal; “GNKLDLYGKVD” is 29-39aa and the signal is labeled with yellow; “NFMQQRGNGFATYRNTD” is 140-156aa and is shown by pink signal; “RAETYTGGLKYD” is 234-245aa and its signal is in orange; “VGSFDYGRNYGVVYD” is 106-120aa and shown with cyan color signal; “NQFTRDAGINTD” is 345-356aa and indicated with in green signal; “ANNIYLAAQYTQTYNATRVGSLG” is 246-268aa and its signal is blue; “YKINLLDDNQFTRD” is 337-350aa and labeled with green signals

OmpC is known as an osmotic-responsive outer membrane porin (Hamner et al. 2013). Several OmpC structures of *E. coli* have been published and are available in the PDB database. Further analysis of ECO157 OmpC (Accession: QEQ39178.1) sequences with this *E. coli* OmpC showed that *E. coli* 2IXX had the highest homology (92.37%) and sequence coverage (94%) to ECO157 OmpC, indicating the high similarity among these structures. With the topology information available about 2IXX in the OPM database (Supplemental Figure S2), 14 eluted peptides (Fig. 3A) from group A and eight identified peptides (Fig. 3B) from group B were identified on the OmpC (2IXX) structure. Figure 3A and B reveals that the loop structure of three adjacent peptides, including “ANNIYLAAQYTQTYNAT**R**VGSLG” (246-268aa, blue, loop1), “FGLRPSLAYLQ- S**K**G**K**NLGVING**R**NYD” (286-311aa, red, loop2), and “Y**K**INLLDDNQFT**R**DAGINTD” (337-356aa, green, loop3), were exposed on the surface of the outer membrane. The other peptides were buried in the phospholipid bilayer. Figure 3C, D, and E shows the MS/MS spectrum of the three long peptides: loop1 (blue), loop2 (red), and loop3 (green).

### Epitope mapping of 2G12 against ECO157 OmpC and its sequence homology

**Fig. 4:**
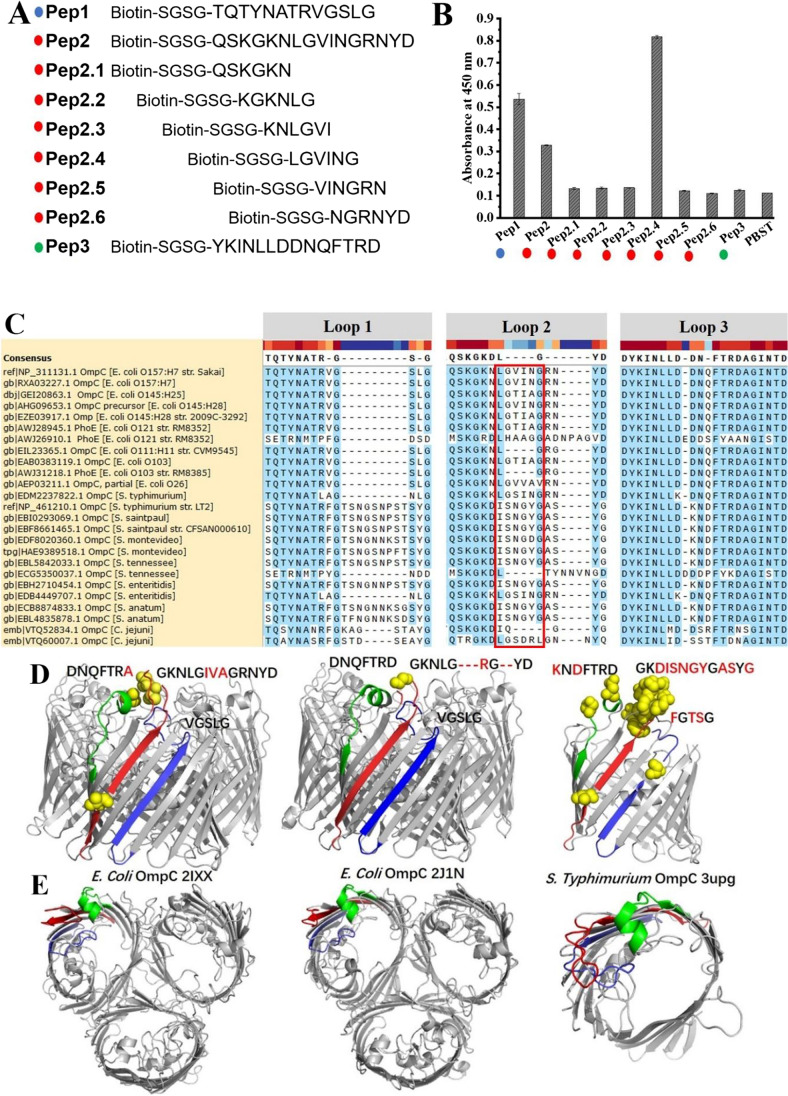
**A** The overlapping peptide library consists of extracellular loops 1, 2, and 3; **B** epitope mapping by indirect ELISA based on the reaction between antibodies and peptides. Higher absorbance indicates higher binding rate of antibody with the corresponding peptide. **C** The sequence homology of three extracellular loops of ECO157 OmpC with other tested non-O157 strains; the dotted line indicates that there is no amino acid; the red box shows the high variance of aminol acids in “LGVIN” of loop2 between strains. The sequence variance (**D**) and orientation difference (**E**) of these three extracellular loops on OmpC structures of general *E. coli* (2IXX, 2J1N) and *S.* Typhimurium (3UPG)

A peptide library of the three extracellular loops was synthesized to study the exact mAb 2G12 epitope. Especially, six overlapping peptides of loop 2 that showed the highest precursor signal in group B were synthesized at the length of 6 with offsets of 2 (Fig. 4A). Results from epitope mapping by indirect ELISA (Fig. 4B) showed that positive reactions were observed with the short peptide 2.4 (LGVING), the long-form of peptide 2 (QSKGKNLGVINGRNYD), and peptide 1 (TQTYNATRVGSLG). These results showed that the extracellular parts of loop 2 and loop 1 were recognized by mAb 2G12.

To better understand the specificity of mAb 2G12 for ECO157, we further investigated the sequence homology of the three extracellular loops with other tested non-O157 strains that are available in NCBI. These non-O157 strains did not react with mAb 2G12 at 6 log_10_ CFU/mL in our previous study (Wang et al. 2021). Results in Fig. 4C show that the presented sequences of loop 1 (TQTYNATRVGSLG) and loop 3 (YKINLLDDNQFTRD) in 10 out of 11 *E. coli* strains shared 81.82% identity and were conservative (Lipman et al. 2002). Notably, the dotted line in Fig. 4C indicates no amino acid present, and their sequences were identical. By contrast, the sequence of loop 2 (QSKGKNLGVINGRNYD) from ECO157 OmpC was observed with 4–6 amino acid substitutions in the “LGVING” part compared with the other tested non-O157 strains that did not react with mAb 2G12. The unpresented sequence of the eluted peptides corresponding to loops 1, 2, and 3 were also conservative. Further comparison of these three extracellular loops with the OmpC of other non-O157 bacteria (homology ranging from 62 to 90% in overall BLAST) showed similar results (Supplemental Figure S3), with both loop 1 and loop 3 being conservative and the “LGVING” part of loop 2 highly variable. Analysis of the published OmpC structures of *E. coli* (2IXX, 2J1N) and *S*. Typhimurium (3UPG) showed that the sequence difference (yellow dots) of ECO157 from these strains was mainly located on loop 2 (Fig. 4D). Figure 4E indicates that the structural differences of the three extracellular OmpC loops from *E. coli* strains and *S.* Typhimurium were mainly attributed to the sequence, length, and position of loop 2. These results revealed that the “LGVING” part of loop 2 on OmpC was the dominant part that contributed to the specificity of mAb 2G12 of ECO157.

### The availability of epitopes under different simulated stressful conditions

**Fig. 5:**
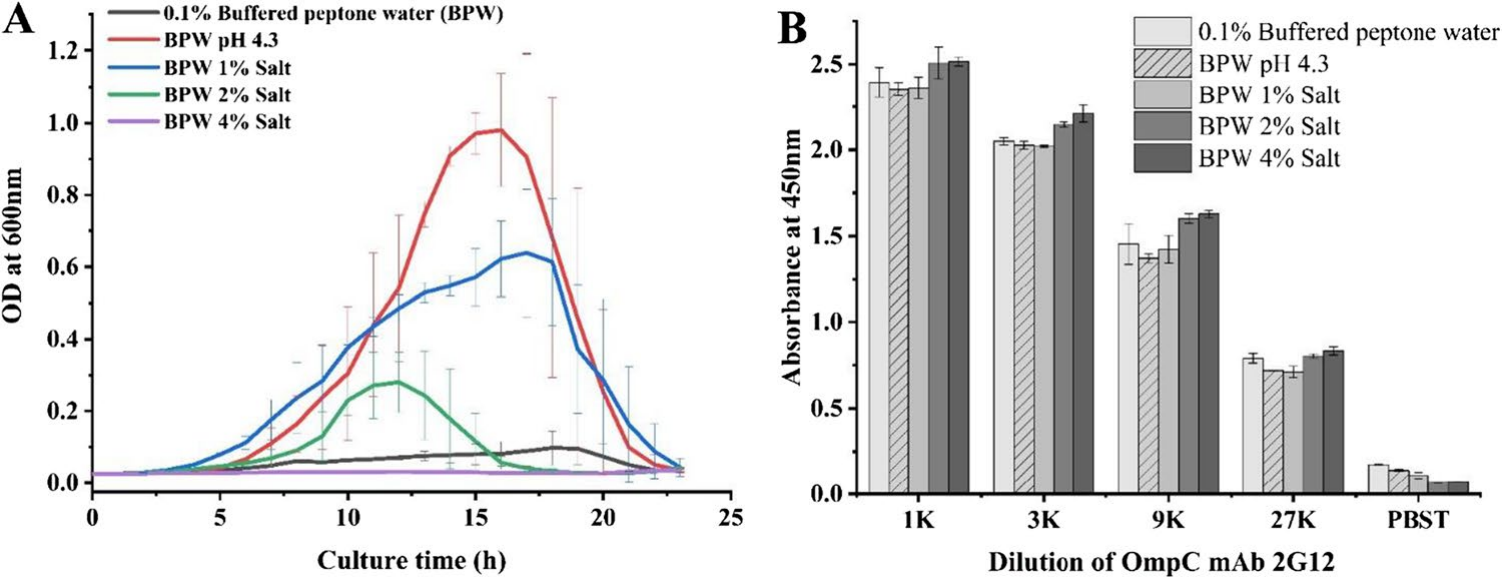
**A** The growth curve of ECO157 (indicated by OD values) under different osmotic pressure, low nutrition level, and low pH level in buffered peptone water (PBS). **B** The binding of OmpC mAb 2G12 against ECO157 grown in different osmotic pressure, low nutrition level, and low pH level in buffered peptone water; the ECO157 was coated at 7.0 log_10_ CFU/mL on the microplate

The availability of OmpC epitopes when ECO157 is grown in different stressful conditions needs to be evaluated since their availability directly impacts the binding efficacy between ECO157 cells and antibodies, especially pathogens in real food samples are typically under unfavorable conditions and in low concentrations. Figure 5A shows that, compared with the normal BPW group (1% salt, pH 7.2), the growth of ECO157 was inhibited at higher salt concentrations (2% and 4%). Low nutrition levels (which was mimicked by 0.1% (m/v) peptone water) also significantly slowed down the growth. However, BPW with a pH value adjusted to 4.3 did not inhibit the growth of ECO157. The plate counting results in Supplemental Figure S4 show that the inoculated level of ECO157 was 7.17 log_10_ CFU/mL, and the cell concentration of ECO157 in BPW with different growth factors both increased except when it is supplemented with 4% NaCl. The results of whole-cell ELISA with ECO157 (7.0 log_10_ CFU/mL) in Fig. 5B show that, compared with the ECO157 grown in normal BPW (OD_450_ = 2.361 ± 0.064), the binding of mAb 2G12 (1 K, 4 μg/mL) increased as the salt concentrations increased from 1% (OD_450_ = 2.508 ± 0.093) to 4% (OD_450_ = 2.517 ± 0.026). In the meantime, the binding of mAb 2G12 with ECO157 grown in BPW with pH 4.3 (OD_450_ = 2.355 ± 0.040) and 0.1% peptone water (OD_450_ = 2.394 ± 0.088) slightly decreased. These results indicated the good availability and stability of this OmpC epitope for the recognition and binding with mAb 2G12.

## Discussion

Wang et al. (2020) revealed that the newly identified mAb 2G12 had good specificity for recognizing ECO157 and it bound with a high abundance protein of approximately 35 kDa. In addition, mAb 2G12 bound with an approximately 70 kDa protein of ECO157 CICC 21,530. However, little is known about the identity of these proteins. In this study, the target protein and the epitope of mAb 2G12 were first purified and isolated for better understanding its specificity at the protein and sequence levels. The purification and quantification of the protein by acetone precipitation, 2D clean-up kit, and 2D quant-kit were essential to remove nucleic acids, polysaccharides, and salt, which usually cause smears on the 2-DE gel (Görg et al. 2004). The size of the OmpC was slightly larger than 35 kDa (pI at around 4.5) based on its position on the 2-DE or SDS-PAGE gel. This location on the 2-DE gel was comparable with the proteomics of *E. coli* reported by Molloy (Molloy et al. 2000). Based on MALDI-TOP-MS, the size of OmpC was 40.48 kDa (pI 4.5). The size differences seen between the SDS-PAGE result and the MALDI-TOF–MS results were expected as previous studies have shown the estimation of protein MW by SDS-PAGE might not be as precise as MALDI-TOF–MS (Liu et al. 2010). Chaperone DnaK located in the peripheral or inner membrane was detected in WB as protein samples were purified directly from whole-cell lysates were used, and chaperone DnaK is involved in binding to nascent or unfolded polypeptides (Calloni et al. 2012). The direct identification of the target protein band (~ 35 kDa) of ATCC 35,150 in the SDS-PAGE gel by in-gel digestion and the LC–MS/MS also showed that the leading protein was OmpC (Supplemental Table S3). As reported previously by Wang et al. (2021), the 70 kDa band was only observed for ECO157 CICC 21,530 and the ~ 35 kDa band was mainly from other ECO157 strains. All of these above results revealed that OmpC was the target outer membrane protein of mAb 2G12.

The epitope excision strategy of IAC-based affinity mass spectrometry was successful in our study. The decreased bound peptides on the column with more trypsin (used in group B) indicated more thorough hydrolysis and more efficient identification of candidate target peptides (Supplemental Tables S1 and S2). Trypsin cleaves exclusively C-terminal to arginine (R) and lysine (K) residues except when they are followed by a proline (P) (Olsen et al. 2004). The residue cleavage sites in these peptides suggested that they were protected from hydrolysis by the mAb in groups A and B. The peptide (red) with a higher precursor signal (1269.36) in group B after IAC and LC–MS/MS (Fig. 3D) indicated a relatively higher residue amount in the column before elution, and it was well protected by the mAb. Our results also showed that a theoretical molecular ratio of enzyme to protein at 2:1 for 30 min (group B) was essential to ensure thorough cleavage of protein during IAC. The in situ digestion with the optimal amount of trypsin in IAC columns was the key to cleave the unrelated peptides and protect the epitope from hydrolysis (Table 1). In the previous work, the in situ digestion of recombinant PD-L1 protein in IAC with molecular ratio of trypsin to protein at 1.63:1 also successfully identified the epitope resides (Hao et al. 2015).

To simplify the sample preparation, a protein cocktail purified from ECO157 cell lysates was used for IAC in this study. In many previous epitope identification studies, recombinant protein samples were usually used (47 out of 52 studies), although native protein (3 out of 52 reports) and cell lysates (2 out of 52 reports) were also adopted (Opuni et al. 2018; Rinaldi et al. 2019). The detection of some impurities like lpp (8.3 kDa, pI 9.3) and OmpA (37.2 kDa, pI 5.99) with nonmatched pI values and MWs in this study indicated that there was non-specific binding with the relatively complex cell protein samples, which may complicate the analysis of results from affinity mass spectrometry. This potential drawback was overcome by two critical steps in our epitope identification. The first one was that the target protein was identified by 2-DE, WB, and MALDI-TOF/TOF before affinity mass spectrometry. By doing so, the target protein was revealed to be the OmpC on the cell outer membrane. The other step was that, with more trypsin added in group B and a more thorough digestion, the number of detected OmpC peptides and spectra ranked first among the all the peptides and spectra.

Fourteen eluted peptides in group A and eight eluted peptides in group B (ranged from 11 to 25aa) after LC–MS/MS challenged the epitope mapping because of the need for a relatively large overlapping peptide library. The location of these peptides on the highly homogenous OmpC structure and the topology information clearly showed that only three peptides on the extracellular side of the outer membrane were available for antibody binding. This was based on the fact that many Omps are inserted into the cell membrane, and only the extracellular parts are involved in the interaction with the host cell and antibodies (Noinaj et al. 2013; Rollauer et al. 2015). Protein structure and topology information identified in this study were further confirmed by information obtained from the PDB and OPM databases. In addition, our results match well with the previous report by Lou et al. (2011). In this study, the experimentally determined crystal structures of five OmpC (2XE1, 2XE2, 2XE3, 2XE5, 2XG6) from clinical *E. coli* mutants were very close to the OmpC structure (7JZ3) of *E. coli* K12. These structures also showed that there were eight extracellular loops on OmpC while three of the eluted peptides in our study located on the extracellular loops as well.

The epitope mapping results by indirect ELISA showed that the OmpC mAb 2G12 reacted with the synthesized peptides of loop 2 (LGVING) and loop 1 (TQTYNATRVGSLG). Both were modified with spacer-arm-linked biotin at the N terminal and attached to the avidin-coated microplate. The absorbance with the extracellular parts of loop 2 (OD_450_ = 0.817 ± 0.011) was higher than loop 1 (OD_450_ = 0.536 ± 0.009). Because of the wide distribution of the OmpR/EnvZ two-component system in bacteria, OmpC is conservative between different serotypes of *E. coli* and other Gram-negative bacteria. The sequence and the structural base for the specificity of mAb 2G12 were further checked by sequence BLAST with strains not reacting with mAb 2G12. As shown in Fig. 4C, the extracellular part of loop 1 was conservative in many of the tested *E. coli* strains, while the “LGVING” part of loop 2 showed high amino acid variance. The same results were obtained in the overall BLAST analysis of OmpC sequences of all the non-O157 strains with clear classification at the species level (Supplemental Figure S3). Therefore, the specificity of mAb 2G12 was largely attributed to the more accessible and specific peptide “LGVING” on loop 2.

The overall BLAST of the target peptides with non-tested strains found that *Shigella* strains and *E. coli* O145 strains have the identical sequence of “LGVING” on loop 2 (Supplemental Figure S3). This indicates possible cross-reaction between these strains with mAb 2G12. However, our previous whole-cell ELISA study of three *Shigella* strains did not show any reaction with mAb 2G12 and an *E. coli* O145 strain weakly reacted with mAb 2G12 (Wang et al. 2021). The antibody specificity against bacteria cells is determined by many factors, such as the physical barrel of outer polysaccharides (Storek et al. 2018). According to a recent report by Domínguez-Medina (Domínguez-Medina et al. 2020), the structure difference of the O antigen resulted in different pore sizes and structures above the porin protein, which restricted IgG binding with Omp even when there was only a single amino acid residue substitution. Therefore, the antibody specificity was shaped by the sequence specificity of the two extracellular loops and further improved by the physical barrel of outer polysaccharides.

Internal and external factors associated with food or food processing environment pose many stresses to bacteria and may alter the expression of surface receptors (Hahm and Bhunia 2006). These surface changes could, in turn, impact the binding affinity of antibodies and the recovery of viable bacterial cells in pre-analytical sample processing (Dwivedi and Jaykus 2011). For example, starved ECO157 incubated in water for a long period resulted in the loss of O antigens in many cells, suggesting that immunologic methods based on the O157 O antigen may not be able to isolate the verocytotoxin-producing *E. coli* under this condition (Hara-Kudo et al. 2000). *E. coli* incubated in low (3.5) or high (9) pH for 24 h has been shown to produce a much smaller signal when detected with a bead-based immunoassay (Denes and Wiedmann 2014). After 6 h and 24 h exposure to the combined stresses of pH 5.5 and 3.5% NaCl at 4 °C, antibody reactions to ECO157 respectively showed 33% and 17% reductions (Hahm and Bhunia 2006). Previous studies reported that the expression of OmpC could be regulated by osmotic stresses, pH values, temperatures, nutrient levels, and the gut environment (Kojima and Nikaido 2014; Lin et al. 2002; Özkanca and Flint 2002; Sleator and Hill 2002; Thomas and Booth 1992).

In this study, to analyze the availability of the epitope with mAb 2G12 under different stressful environments in food, the clinical ECO157 isolate LJH643 obtained from a cantaloupe outbreak was used because it is directly related with food poisoning. Our results showed that binding of OmpC antibody with ECO157 slightly increased when the bacteria were grown in an environment with higher osmolarity and slightly decreased when grown in an environment with lower pH and nutrition level, which indicated that the expression and availability of the OmpC epitope were relatively stable for the wide application of immunomagnetic separation and immunoassays.

In summary, mAb 2G12 have shown good specificity to ECO157. The target protein and epitope of mAb 2G12 were identified by combining 2-DE, WB, MALDI-TOF/TOF, affinity mass spectrometry, epitope mapping, and protein structure, topology, and sequence homology analysis. The epitope consists of two extracellular loops of OmpC, the ECO157-specific “LGVING,” and an adjacent “TQTYNATRVGSLG.” “TQTYNATRVGSLG” was found to be more conservative than “LGVING.” The ECO157-specific extracellular loop with the peptide “LGVING” was the dominant part contributing to antibody specificity. The availability and accessibility of “LGVING” is stable when ECO157 is grown under various food environment conditions/stresses. The loop structure with “LGVING” present on OmpC serves as a promising target for the preparation of ECO157 diagnostic antibodies with good specificity. Furthermore, the affinity mass spectrometry and protein topology approaches were proven to be efficient methods for studying epitopes and their reactions with antibodies.

## Supplementary Information

Below is the link to the electronic supplementary material.

Supplementary file1 (PDF 1715 KB)

## Data Availability

All major data generated and analyzed in this study are included in this manuscript and its supplementary information files. Other data is available from the first and corresponding authors on reasonable request.
