# Supplementary material for: Identification of a specific surface epitope of OmpC for Escherichia coli O157:H7 with protein topology facilitated affinity mass spectrometry
Source: Appl Microbiol Biotechnol. 2021 Aug 25;105(18):6819–33. doi: 10.1007/s00253-021-11511-8 (PMC8426304; doi:10.1007/s00253-021-11511-8)
Supplement: Supplementary file 1 — Supplementary file1 (PDF 1715 KB) [file 253_2021_11511_MOESM1_ESM.pdf]

Journal name: Applied Microbiology and Biotechnology

Identification of a specific surface epitope on OmpC for *Escherichia coli* O157:H7 detection with protein topology facilitated affinity mass spectrometry

Wenbin Wang [a,b,c*], Xinyue Zhou[a,b], Yunong Sang [a,b], Xiaxia Liang[a,b], Jianxin Liu [a,b], Saikun Pan [a,b,c], Luxin Wang[d*]

a, Jiangsu Key Laboratory of Marine Bioresources and Environment, Jiangsu Ocean University, Lianyungang, Jiangsu, China

b, Co-Innovation Center of Jiangsu Marine Bio-industry Technology, Jiangsu Ocean University, Lianyungang, Jiangsu, China

c, Jiangsu Key Laboratory of Marine Biotechnology, Jiangsu Ocean University, Lianyungang, Jiangsu, China

d. Department of Food Science and Technology, University of California Davis, Davis, CA 95618, USA

** Author to whom correspondence should be addressed*

Tel: +86-518-8589 2122, Fax: +86-518-8589 5429

mail: lxwang@ucdavis.edu; wenbin66@jou.edu.cn

Table S1. Peptides of OmpC identified with 260μg trypsin (group A) after IAC and LC-MS/MS.

Number	Score	Accession	Gene	Mw(kD)	Conf	Sequence	Location (aa)	dMass	Prec MW	Prec m/z	Theor MW	Theor m/z	Theor z	Spectrum	Time	Precursor Signal
1	27.46	Q8XE41	*ompC*	40.51	99.00	AEVYNKDGNKLDLYGKVD	22-39 (yellow)	0.0106748	2040.0271	681.0163	2040.016357	681.012756	3	1.1.1.2001.17	15.8129	1706.496
2	27.46	Q8XE41	*ompC*	40.51	99.00	ANNIYLAAQYTQTYNATRVGSLG	246-268 (blue)	0.0197237	2487.26928	1244.642	2487.250732	1244.63256	2	1.1.1.2117.21	18.9692	1054.476
3	27.46	Q8XE41	*ompC*	40.51	99.00	FGLRPSLAYLQSKGKNLGVINGRNYDD	286-311 (red)	0.01808720	2994.58569	749.6537	2994.567627	749.649169	4	1.1.1.2083.13	18.0223	1839.844
4	27.46	Q8XE41	*ompC*	40.51	99.00	GLNFAVQYQGKNG	163-175 (palegreen)	0.00480712	1394.69909	698.3568	1394.694214	698.354431	2	1.1.1.2062.12	17.4403	776.7463
5	27.46	Q8XE41	*ompC*	40.51	99.00	GLNFAVQYQGKNGSVSGEGMTNNGREALRQNGD	163-195 (palegreen)	0.0281616	3467.65185	867.9202	3467.623535	867.913208	4	1.1.1.2028.17	16.541	8221.004
6	27.46	Q8XE41	*ompC*	40.51	99.00	GNKLDLYGKVD	29-39 (yellow)	0.00318184	1220.64331	611.3289	1220.640137	611.327331	2	1.1.1.2001.15	15.8112	1297.818
7	27.46	Q8XE41	*ompC*	40.51	99.00	GVGGSITYDYEGFGIGAAVSSSKRTD	196-221 (wheat)	0.01179	2593.24145	865.4211	2593.229736	865.417175	3	1.1.1.2114.10	18.8771	2817.707
8	27.46	Q8XE41	*ompC*	40.51	99.00	TYGSDNFMQQRGNGFATYRNTD	135-156 (pink)	0.0279663	2542.12109	848.381	2542.093262	848.371643	3	1.1.1.2032.11	16.6413	459.0881
9	27.46	Q8XE41	*ompC*	40.51	99.00	VGSFDYGRNYGVVYD	106-120 (cyan)	0.00827876	1709.77685	855.8957	1709.768555	855.891540	2	1.1.1.2086.16	18.1086	1416.839
10	27.46	Q8XE41	*ompC*	40.51	99.00	YGRNYGVVYDVTSWTD	106-126 (cyan)	0.0123537	1893.86572	947.9401	1893.853394	947.93396	2	1.1.2140.16	19.6054	1995.169

11	27.46	Q8XE41	*ompC*	40.51	96.34	YKINLLDDNQFTRDAGINTD	337-356 (green)	0.00681071	2325.13061	776.0508	2325.123779	776.048522	3	1.1.1.2126.9	19.2094	1616.575
12	27.46	Q8XE41	*ompC*	40.51	95.75	NQFTRDAGINTD	345-356 (green)	0.0147924	1350.63122	676.3229	1350.616455	676.315490	2	1.1.1.1992.2	15.5554	64.1142
13	27.46	Q8XE41	*ompC*	40.51	95.61	GNKLDLYGKVDGLHYFSD	29-46 (yellow)	0.0101977	2040.00549	681.0091	2039.995239	681.005676	3	1.1.1.2091.9	18.2411	2282.842
14	27.46	Q8XE41	*ompC*	40.51	99.00	GNKLDLYGKVD	29-39 (yellow)	-1.71630001	1218.92382	610.4692	1220.640137	611.327331	2	1.1.1.2002.14	15.838	503.0494

Table S2. Peptides of OmpC identified with 520μg trypsin (group B) after IAC and LC-MS/MS.

Number	Score	Accession	Gene	Mw(kD)	Conf	Sequence	Location (aa)	dMass	Prec MW	Prec m/z	Theor MW	Theor m/z	Theor z	Spectrum	Time	Precursor Signal
1	17.58	Q8XE41	*ompC*	40.51	99.00	FGLRPSLAYLQSKGKNLGVINGRNYD	286-311 (red)	0.0167099	2879.5574	720.8966	2879.5405	720.89246	4	1.1.1.2109.16	18.0951	1269.363
2	17.58	Q8XE41	*ompC*	40.51	99.00	GNKLDLYGKVD	29-39 (yellow)	-0.0040203	1220.6361	611.3253	1220.6401	611.32733	2	1.1.1.2030.4	15.9221	405.3083
3	17.58	Q8XE41	*ompC*	40.51	99.00	NFMQQRGNGFATYRNTD	140-156 (pink)	0.0020208	2034.8987	679.3068	2034.8966	679.30615	3	1.1.1.1979.3	14.6853	195.7011
4	17.58	Q8XE41	*ompC*	40.51	99.00	RAETYTGGLKYD	234-245 (orange)	-0.0014321	1372.6609	687.3377	1372.6624	687.33844	2	1.1.1.1993.2	14.9236	124.9317
5	17.58	Q8XE41	*ompC*	40.51	99.00	VGSFDYGRNYGVVYD	106-120 (cyan)	-0.0002662	1709.7683	855.8914	1709.7686	855.89154	2	1.1.1.2111.17	18.151	450.5602
6	17.58	Q8XE41	*ompC*	40.51	99.00	NQFTRDAGINTD	345-356 (green)	0.0012426	1350.6177	676.3161	1350.6165	676.31549	2	1.1.1.2019.4	15.6182	72.3196
7	17.58	Q8XE41	*ompC*	40.51	99.00	ANNIYLAAQYTQTYNATRVGSLG	246-268 (blue)	0.0006807	2487.2515	1244.633	2487.2507	1244.6326	2	1.1.1.2143.21	19.0217	540.2604
8	17.58	Q8XE41	*ompC*	40.51	99.00	YKINLLDDNQFTRD	337-350 (green)	0.0022917	1753.8658	585.6292	1753.8635	585.62842	3	1.1.1.2123.3	18.4704	697.079

Table S3. Leading proteins in the target band (approximately 35 kDa) of ECO157 ATCC 35150 identified by the LC-MS/MS after in-gel digestion.

Number	Score	Accession	Gene	Mw(kD)	Subcellular location	Peptides (95%)	Spectra
1	56.56	Q8XE41	*ompC*	40.508	Cell outer membrane	45	140
2	51.02	P0A6P1	*tsf*	30.423	Cytoplasm	30	51
3	50.27	P0A9B2	*gapA*	35.532	Cytoplasm	35	132
4	42.08	P0ABK5	*cysK*	34.49	cytosol	26	81
5	40.73	P0A870	*talB*	35.219	Cytoplasm	24	27
6	39.81	P0A910	*ompA*	37.201	Cell outer membrane	29	147

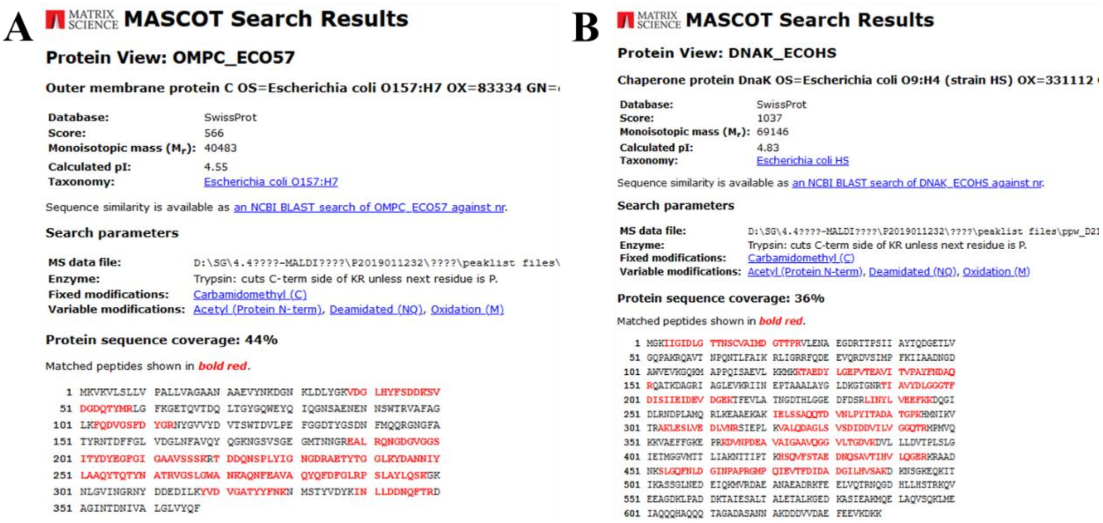

Figure S1. (A) MALDI-TOF/TOF results of protein spots with approximately 35 kDa and pI around 4.5. (B) MALDI-TOF/TOF results of protein spots with approximately 70 kDa and pI around 5.0.

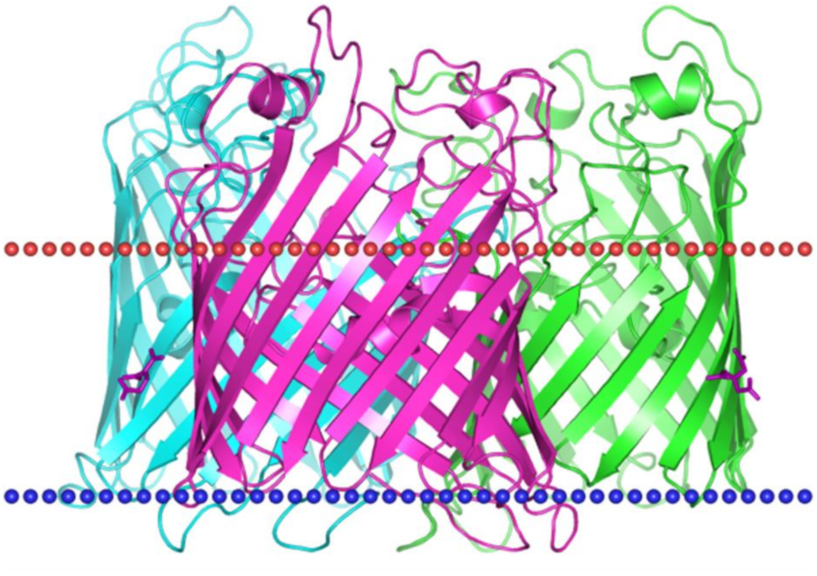

Figure S2. The protein topology of *E. coli* OmpC (2IXX) on the cell outer membrane obtained from the OPM database. The space above the red dotted line indicates the extracellular side of the cell, while the space under the blue dotted line represents the periplasmic side of the cell.

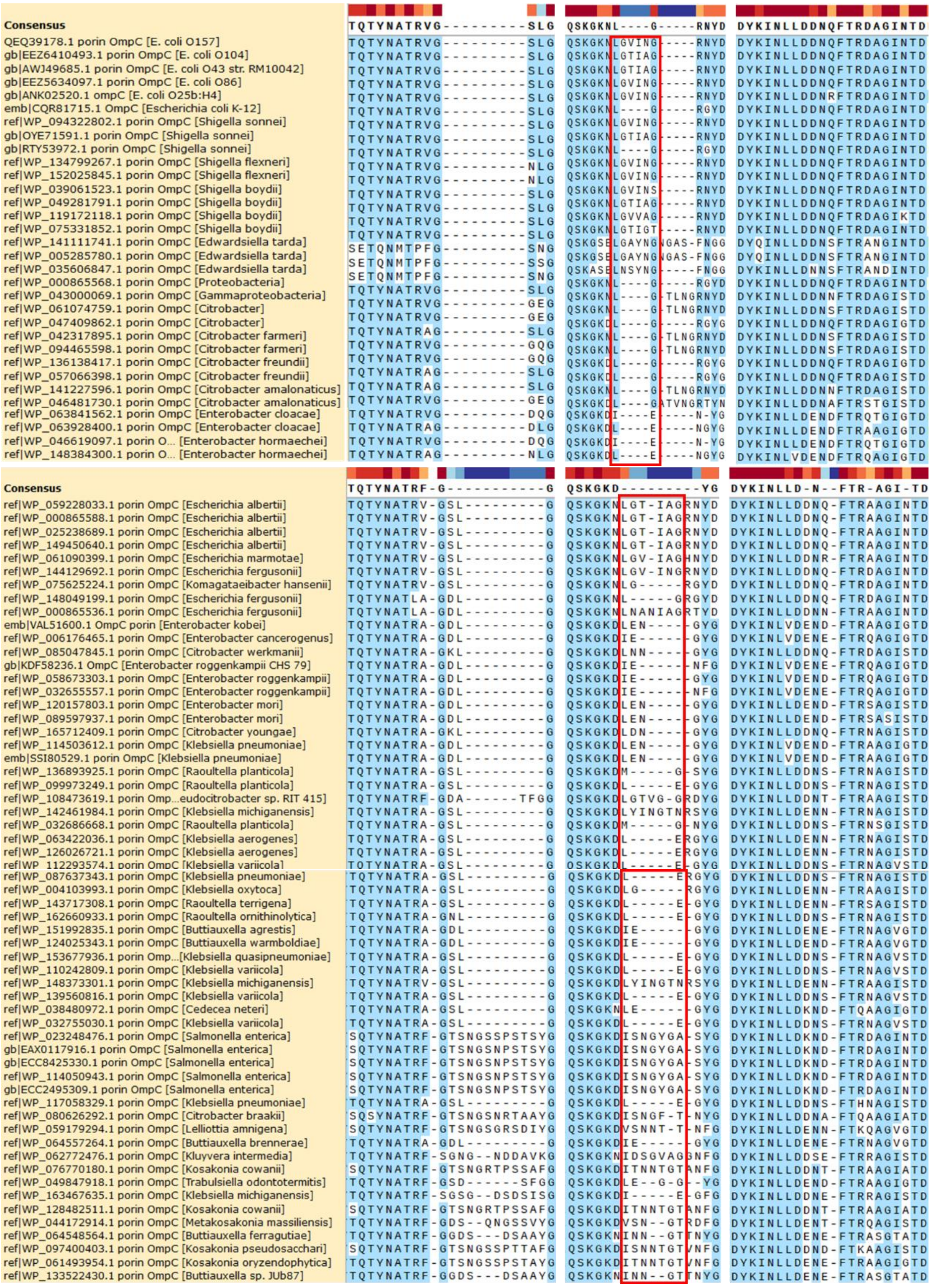
Consensus
TQTYNATRVG---------SLG QSKGKNL----G-----RNYD DYKINLLDDNQFTRDAGINTD
Consensus
TQTYNATRF-G----------G QSKGKD---------YG DYKINLLD-N--FTR-AGI-TD

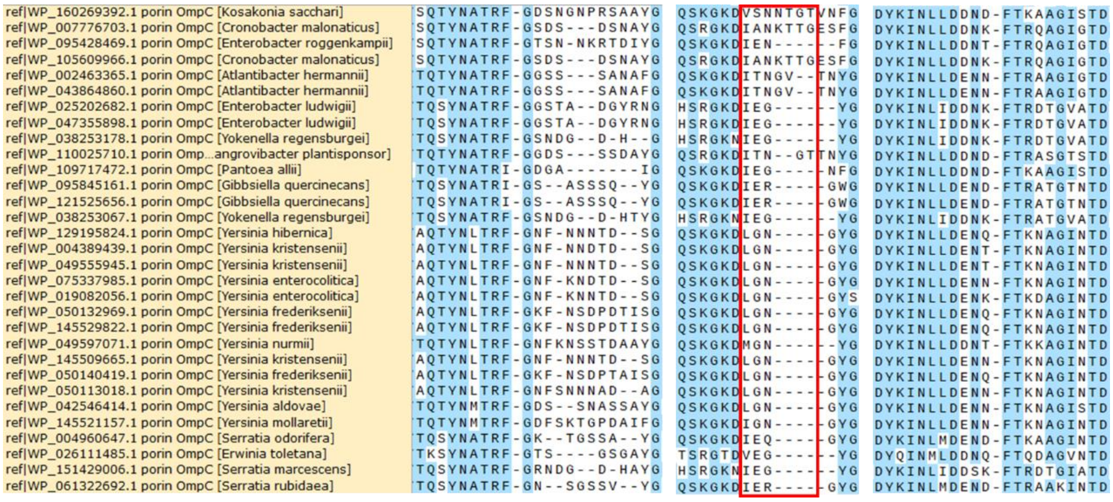

Figure S3. Comparison of the sequence homology of three extracellular loops of OmpC between *E. coli* O157: H7 and 100 strains that have not been tested for cross-reaction with mAb 2G12. The red box highlights the high variance of aminol acids at “LGVIN” of loop2.

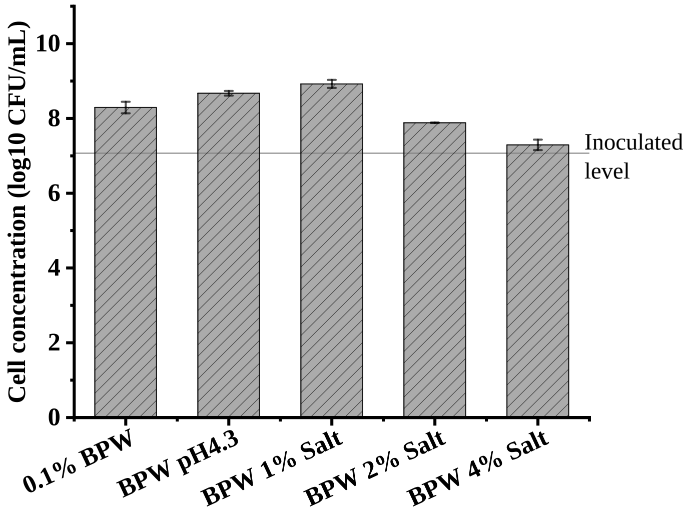

Figure S4. Cell concentrations of ECO157 in 0.1% (m/v) buffered peptone water (BPW), BPW with 1% Salt (pH 7.2), BPW with 2% NaCl, BPW with 4% NaCl, BPW with pH 4.3 (adjusted by HCl) after inoculation with the seed culture at the ratio of 1% (V/V) and incubated at 37 °C for 24 h.
